# Considerations for Physicians Treating Skin of Color: A Narrative Review

**DOI:** 10.7759/cureus.74443

**Published:** 2024-11-25

**Authors:** Hana Elhassan

**Affiliations:** 1 Dermatology, Lauriston Building, Edinburgh, GBR

**Keywords:** healthcare outcomes, health inequality, medical dermatology, medical education, skin of color

## Abstract

Clinical research and medical education regarding dermatoses in people with skin of color (SoC) have historically been neglected, which has played a role in physicians lacking confidence and competence in recognizing and managing dermatoses in diverse cohorts. This has contributed to poorer outcomes in people with SoC in objective domains, such as disease severity indices and prognoses, and subjective domains, such as patient-reported quality-of-life indices. In the first instance, physicians should understand the basic physiological differences underpinning SoC diversity and its clinical implications. This narrative review provides an introductory evidence-based discussion on clinically important topics relating to SoC aimed at non-dermatologists. Topics covered include the cellular basis underpinning skin color diversity and how this affects photodermatoses and malignancies, as well as an overview of how scarring and inflammatory conditions may present differently in people with SoC.

## Introduction and background

'Skin of color' (SoC) refers to a wide range of more darkly pigmented complexions [1]. The term coexists alongside racial and ethnic identifications [1]. It is important not to conflate SoC categorizations with race or ethnicity, given that a vast degree of skin type heterogeneity exists within each race/ethnicity, and race has been found to correlate poorly with skin phototype [2]. Race and ethnicity are both social constructs. Race classifies people based on physical characteristics such as skin color, hair, and facial morphology. Ethnicity focuses on cultural factors such as traditions, language, and shared histories. An understanding that particular skin types may predominate within particular races or ethnicities can help clinicians and patients better understand individuals' health needs. Appreciating a patient's skin type alongside their racial and ethnic profile is important, as their skin type and race aid a biomedical assessment of skin, while their ethnic profile may provide insight into cultural practices, beliefs, and health behaviors that affect patients' dermatological health. This is highly context-dependent, and each patient needs to be assessed in their own unique context. By taking into account a person's skin type, race, and ethnicity, physicians can deliver medically precise, culturally competent care, which may then help to minimize health disparities between different patient groups.

The predominance of a Western lens in medical education and research has led to the historic and ongoing underrepresentation of SoC in educational material [3] and clinical studies [4]. This leads to doctors expressing underconfidence [5] in diagnosing and managing dermatoses in people with SoC, while objective evaluations demonstrate a lack of clinical proficiency [5]. Such findings are concerning, given current demographic trends. Notwithstanding that people with SoC account for the majority of skin tones globally, populations in Western settings are becoming increasingly diverse and mixed [6,7]. It is, therefore, increasingly important that doctors are well-versed in understanding the unique presentations and health needs of people with SoC. While multifactorial, lack of physician confidence, knowledge, and competence is speculated to play a role in people with SoC having poorer dermatologic health outcomes [8].

Therefore, this narrative review aims to provide an introductory, evidence-based overview of the physiological differences underpinning SoC, how some conditions manifest differently, and other important clinical considerations for physicians treating SoC.

Fitzpatrick skin phototype classification system

The Fitzpatrick skin phototype classification system is the most commonly used in clinical practice. It was initially formulated to assess white skin tones' sensitivity to ultraviolet radiation (UVR) during phototherapy and initially consisted of four skin types [1]. It was later developed to include two further classes: 'brown' and 'black' [1]. SoC has conventionally been approximated to Fitzpatrick skin types IV-VI [1].

The system has been criticized for several reasons, including its subjectivity [9] and failure to capture the broad spectrum of human color variation [10] due to being Eurocentric. While European populations have the narrowest skin color variation, half of the system's subgroups are variations of white skin tones; the remaining groups encompass the rest of global skin colors [11]. The system, thus, is profoundly limited in accurately reflecting the true breadth of global skin color diversity. It additionally is functionally limited owing to its original aim to classify skin types according to their propensity to burn in response to UV light. This limits its generalizability as an appropriate classification to other skin responses, such as hyperpigmentation and photoaging, where another system may be better suited. Despite this, it continues to be used to universally classify people for clinical problems beyond its original inception.

In response, several other systems have been formulated [12] to assess skin color variation, including the Taylor hyperpigmentation scale [13], the Eumelanin scale [14], and objective scales using colorimetry and spectrophotometry [15], although the latter currently have limited penetration to clinical settings.

## Review

Methods

Medline and Ovid were searched for (non-MeSH) keywords such as 'photoprotection', 'atopic dermatitis', and 'pigmentation', which were combined with searches for terms such as 'skin of color', 'Black', 'Asian', and 'Hispanic'. Synonyms and similar terms were also used, for example, 'eczema' for 'atopic dermatitis'. Both American and British English spellings were searched. Papers were restricted to those published in English and shortlisted based on their abstract and title. Papers were included following reading their full text, and prioritization for inclusion was based on the relevance of the paper.

Physiology of SoC

Skin color is largely determined by melanin [16], alongside other chromophores such as hemoglobin, beta-carotene, and bilirubin. Melanin is produced by melanocytes within membrane-bound melanosomes in the epidermis. Melanocytes produce two different types of melanin: eumelanin and pheomelanin. Eumelanin is a brown-black pigment that is more abundant in darker skin types, while pheomelanin, a yellow-red pigment, is more abundant in fairer skin types. As melanosomes fill with melanin, they are transferred to keratinocytes. The type and amount of melanin and differences in the size and distribution of melanosomes determine skin color [16]. Darker skin types also have larger melanocytes, filled with greater quantities of melanin, which are laid throughout the epidermis and degrade over a longer period of time [16].

The above cellular differences have clinical implications. This distribution of melanin and melanocytes plays a key role in people with SoC having more robust protection from UV-related damage [17]. Furthermore, darkly pigmented skin also poses some diagnostic challenges. Erythema, the 'redness' classically seen in fairer skin types due to increased blood flow in capillaries, usually associated with inflammation, may be absent or appear markedly different in darker skin types. Here, it can manifest as a subtle darkening of the background skin color [18], which we will discuss later. Other features of inflammation should, therefore, be closely examined. In addition to localized pain and heat, skin should be examined for contour changes and signs of swelling [18]. Swelling can be suggested by the widening of skin pores or a greater prominence of skin pores being visible [18].

Scarring

Hypertrophic and keloid scars are abnormal scars produced by aberrant wound healing following trauma. Although keloids can develop in all skin colors, their development skews heavily towards darker skin types [19]. Additional risk factors include female gender, younger age, and particular anatomical sites [20]. People with SoC are thought to be at higher risk of developing hypertrophic scars, but epidemiological evidence underpinning this assertion is weak owing to limited data [21]. Fibroblasts are particularly implicated [19] in scar formation. In SoC, tissue injuries are prone to a more pronounced inflammatory response [20], resulting in these scars forming. In this demographic, keloid and hypertrophic scars can be particularly large and hyperpigmented [20]. SoC has been shown to contain larger and more numerous fibroblasts [22], which may explain the keloid phenomena in this demographic; however, no overt genetic or molecular mechanism has been established to explain this. The cosmetic appearance of these scars can cause significant distress to patients. Additionally, keloids can be symptomatic of itch or pain and can cause functional disability, particularly over joints or mobile sites. Accordingly, clinicians should be mindful of the higher propensity for people with SoC to develop such scars. This is especially true when risk is compounded by the presence of additional risk factors. These cases may benefit from additional experienced input to minimize adverse outcomes in these patients.

Pigment disorders and photodermatoses

People with SoC have a greater proportion of eumelanin to pheomelanin and more melanin in the upper layers of the epidermis [23], bestowing greater intrinsic photoprotection. This includes reduced photocarcinogenesis risk. However, they remain at risk of skin damage caused by UVR [24].

SoC is particularly at risk of photodermatoses and photoaging. Some photodermatoses, such as polymorphic light eruptions and chronic actinic dermatitis, are more common in people with SoC [25]. Visible light accounts for 50% of solar radiation and has been shown to impact pigmentary disorders [26]. SoC is more susceptible to photodamage from visible light. Additionally, high-energy visible light covering the violet to blue-light wavelengths has been shown to promote pigmentation in Fitzpatrick skin types III to VI but not those with lighter skin [26].

People with SoC are additionally more prone to developing pigment disorders such as postinflammatory hyperpigmentation (PIH) and melasma [27] as a result of photo exposure. This impact should not be overlooked, as dyschromia from PIH and melasma account for the second most common reason for people with SoC to consult dermatologists [28], with the lesions negatively impacting patients' quality of life [29].

With regards to treating photoaging effects, some authors have cautioned against the use of chemical peels and ablative lasers in people with SoC, as they are at increased risk of developing PIH from these modalities [30].

Photoprotection

SoC has inherent photoprotective properties, as melanin diminishes the effects of UVR. In a cadaveric study, the transmission of UVA and UVB through the black epidermis was one-third and a quarter of that of the white epidermis, respectively [17]. The black epidermis is estimated to have a sun-protective factor (SPF) of 13.4 against UVB, compared to 3.4 in the white epidermis [17]. As outlined above, people with SoC are, however, more prone to particular photodermatoses. Photoprotection is, therefore, still vitally important. Worryingly, physicians are less likely to consider and either counsel on or prescribe sunscreen to people with SoC [31].

Very broadly speaking, SoC populations have generally been found to be diligent with sun avoidance and wearing sun-protective clothing but have consistently been shown to have poorer uptake of sunscreen use [32-35]. Social and cultural contexts have been implicated. Sun protection and sun avoidance are commonly practiced in areas of East Asia, largely attributed to cultural preferences for fairer skin, especially among women [36,37]. In contrast, cultural integration of Hispanic populations in the US has been associated with a reduced likelihood of sun-protective behaviors, including wearing sun-protective clothes and seeking shade [38]. This highlights the importance of paying regard to patients' personal values, environment, and culture, as well as their skin type, when counseling on photoprotective measures.

It is important that the underuse of sunscreen in people with SoC is addressed. People with SoC, and potentially some physicians, may perceive that sunscreen is unnecessary due to some understanding that people with darker skin are more protected against UVR [33,34]. Educational interventions need to be appropriately planned to take into account the social contexts within which people with SoC are situated [39]. Some evidence suggests that messaging on the importance of both cosmetic protection and avoidance of skin cancer resonates, but the former is more effective in initiating photoprotective behaviors and the latter for cementing them [40].

One barrier to sunscreen use that particularly affects people with SoC is that active ingredients in some sunscreens, such as zinc oxide and iron oxide, leave a white residue on the skin. This is significantly less appealing to people with SoC and can impact compliance [41]. The use of tinted sunscreens may help alleviate this issue as this promotes cosmesis, especially in those with SoC pigmentary disorders, while also providing photoprotection to prevent further exacerbation of pigmentary disorders.

Skin cancers

While the photoprotective properties of melanin help result in lower incidences of skin cancer in SoC [42], it remains a concern. Although people with SoC have reduced rates of melanoma, mortality rates associated with melanoma in this demographic are disproportionately greater than those of white individuals [43,44]. These disparities in mortality may be due to the underuse of dermatologic care in SoC populations [45], resulting in delayed diagnosis. In contrast to lighter skin demographics, in SoC, melanoma has a predilection for non-sun-exposed sites, including the palms and soles. This may delay detection, particularly as there may be associated difficulties with patients self-examining these areas [46], therefore presenting later to their medical provider. The variation in pigmentary appearances in melanoma, basal cell carcinoma (BCC), and squamous cell carcinoma (SCC) may further increase misdiagnosis [47]. For example, in SoC, BCCs can present as pigmented nodular or scar-like lesions or as pigmented or erythematous patches or plaques. They can mimic a plethora of other conditions, including sebaceous keratoses, benign naevi, dermatosis papulosa nigra, but also melanoma and SCC, making the diagnosis particularly challenging [48]. In summary, the relatively lower prevalence and wider clinical variability of the presentation of carcinomas in SoC make diagnosis particularly challenging.

Atopic dermatitis (AD)

AD is exceedingly common and affects all racial groups [49]. It significantly affects patients' quality of life, including intrusion of sleep, with people with SoC being worse affected [50,51]. It is particularly relevant to people with SoC, as it is twice as common among children of Black, East Asian, and South Asian races [52-55] compared to White populations; one center found it accounts for 29% of all dermatological diagnoses made in Black and Asian children and 13% in White children [49]. A significant proportion of AD diagnoses are classed as 'atypical', falling outside established case definitions [50]. Classic skin changes in AD include erythema, but this can be challenging to note in highly pigmented skin, as previously outlined, and evaluation for other clinical signs may be required. Instead of a red-pink shade, active AD can present in brown, purple, or ash-gray shades [50].

There are some additional differences in how AD presents with respect to morphology and distribution in darker skin types compared to lighter skin types. In people with SoC, AD more commonly presents on the extensor surfaces [50,56], with perifollicular accumulation and dorsal lichenoid plaques [56] being more common.

When assessing AD, clinicians often look for signs of redness, its severity, and distribution and use this to guide management. This features in the eczema area and severity index (EASI) score. Due to the differences in clinical presentation of AD in people with SoC, accurate clinical assessment of disease severity can be challenging, and disease severity in these populations is often underestimated [57], with resultant suboptimal management. To compensate, some suggest increasing EASI erythema scores by 1 in people with SoC [58] or replacing the erythema component of the EASI with other descriptors of skin changes, including those associated with active AD in people with SoC [59].

Given the prevalence of AD and its significant impact, it is important that clinicians are aware of these differences so that they can promptly recognize AD severity in people with SoC. Physicians' unawareness of these signs risks suboptimal management by underestimating disease severity in people with SoC and may even cause misdiagnosis [60].

Psoriasis

There is a lower incidence of psoriasis in people with SoC compared to white skin. In SoC, psoriatic plaques may have varying shades of pink [61,62] and can sometimes appear purple. Plaques are often larger, scalier, and thicker in people with SoC [61,62]. Similar challenges exist with assessing psoriasis severity, as in eczema, as clinicians often look for redness of the skin. This may again lead to misdiagnosis and underestimation of disease severity. This issue is particularly significant for both psoriasis and AD in people with SoC, as they are prone to develop PIH, as described earlier. This can be hugely disfiguring, may persist for years, and be a super-added cause of psychological distress for patients, which is more pronounced in people with SoC [63].

## Conclusions

This review highlights the importance of how being a person with SoC carries clinical significance. The molecular and cellular basis for how diverse skin colors are produced has clinical implications for heterogeneity in the susceptibility, manifestation, and clinical outcomes of many dermatoses. While undergraduate and postgraduate medical curricula catch up to correct historic blindspots on this matter, it is crucial that clinicians practicing today familiarize themselves with the basic principles outlined in this paper to help them deliver both effective and equitable healthcare to people with SoC and help minimize disparities in this group.
